# Randomized Sham-Controlled Pilot Study of Neurocardiac Function in Patients With Acute Ischaemic Stroke Undergoing Heart Rate Variability Biofeedback

**DOI:** 10.3389/fneur.2021.669843

**Published:** 2021-05-26

**Authors:** Timo Siepmann, Paulin Ohle, Annahita Sedghi, Erik Simon, Martin Arndt, Lars-Peder Pallesen, Gerhard Ritschel, Jessica Barlinn, Heinz Reichmann, Volker Puetz, Kristian Barlinn

**Affiliations:** ^1^Department of Neurology, University Hospital Carl Gustav Carus, Technische Universität Dresden, Dresden, Germany; ^2^Department of Psychotherapy and Psychosomatic Medicine, University Hospital Carl Gustav Carus, Technische Universität Dresden, Dresden, Germany

**Keywords:** biofeedback, stroke, cardiac, heart rate variability, parasympathetic, stroke unit

## Abstract

**Background:** Neurocardiac dysfunction worsens clinical outcome and increases mortality in stroke survivors. We hypothesized that heart rate variability (HRV) biofeedback improves neurocardiac function by modulating autonomic nervous system activity after acute ischaemic stroke (AIS).

**Methods:** We randomly allocated (1:1) 48 acute ischaemic stroke patients to receive nine sessions of HRV- or sham biofeedback over 3 days in addition to comprehensive stroke unit care. Before and after the intervention patients were evaluated for HRV *via* standard deviation of normal-to-normal intervals (SDNN, primary outcome), root mean square of successive differences between normal heartbeats (RMSSD), a predominantly parasympathetic measure, and for sympathetic vasomotor and sudomotor function. Severity of autonomic symptoms was assessed *via* survey of autonomic symptom scale total impact score (TIS) at baseline and after 3 months.

**Results:** We included 48 patients with acute ischaemic stroke [19 females, ages 65 (4.4), median (interquartile range)]. Treatment with HRV biofeedback increased HRV post intervention [SDNN: 43.5 (79.0) ms vs. 34.1 (45.0) ms baseline, *p* = 0.015; RMSSD: 46.0 (140.6) ms vs. 29.1 (52.2) ms baseline, *p* = 0.015] and alleviated autonomic symptoms after 3 months [TIS 3.5 (8.0) vs. 7.5 (7.0) baseline, *p* = 0.029], which was not seen after sham biofeedback (SDNN: *p* = 0.63, RMSSD: *p* = 0.65, TIS: 0.06). There were no changes in sympathetic vasomotor and sudomotor function (*p* = ns).

**Conclusions:** Adding HRV biofeedback to standard stroke unit care led to improved neurocardiac function and sustained alleviation of autonomic symptoms after acute ischaemic stroke, which was likely mediated by a predominantly parasympathetic mechanism.

**Clinical Trial Registration:** www.ClinicalTrials.gov, identifier: NCT03865225.

## Introduction

More than 75% of survivors from acute ischaemic stroke develop symptoms due to impaired neural control of organs that are innervated by the autonomic nervous system comprising heart, vasculature and skin (1, 2). Previous analyses of autonomic nervous system integrity following acute ischaemic stroke revealed impairment of neurocardiac function quantified *via* the predominantly parasympathetic measure heart rate variability (HRV) as well as impaired sympathetic perspiratory gland (sudomotor) function indicating dysregulation of both the sympathetic and the parasympathetic branch of the autonomic nervous system with possible predominance of parasympathetic disturbances (3). Autonomic neurocardiac dysfunction is a critical complication of acute ischaemic stroke which causes debilitating symptoms such as orthostatic hypotension and cardiac arrhythmia and is furthermore associated with worsening of functional outcome and increased mortality (4–6). Treatment to improve neurocardiac function is currently not part of standardized acute or rehabilitative stroke therapy, which can largely be explained by a lack of data on therapeutic approaches.

HRV biofeedback is a non-invasive, non-pharmacological procedure, which is based on a metronomic breathing technique to increase parasympathetic activity and thereby HRV with continuous measuring and visualization of HRV in real-time on a computer screen (7). The technique has been shown to improve neurocardiac function in patients with the primarily cardiovascular disorder of coronary artery disease; however, its potential usefulness in the treatment of patients with acute ischaemic stroke is unknown (8).

We tested the hypothesis that short-term HRV biofeedback can be integrated into stroke unit care to improve neurocardiac function and alleviate symptoms of dysautonomia after acute ischaemic stroke.

## Methods

### Study Design and Population

We conducted a randomized, sham controlled, blinded pilot study at a tertiary stroke center in Germany (University Hospital Carl Gustav Carus, Dresden) in 48 female or male Patients with acute ischaemic stroke who were admitted to our stroke unit within 72 h after onset of symptoms.

We included patients between 18 and 90 years of age with evidence of an ischaemic lesion on cranial computed tomography or magnetic resonance imaging. To avoid confounding of autonomic function assessment we excluded patients with history or clinical evidence of autonomic neuropathy, intake of any tricyclic antidepressant within the last 14 days or atrial fibrillation. Furthermore, we excluded patients who were considered incapable of performing biofeedback because of aphasia, cognitive deficits, respirational insufficiency, blindness, deafness, malignant cerebral infarct, indication for treatment at intensive care unit or other physical limitations with resulting inability to participate in HRV biofeedback training.

### Study Protocol

We randomly allocated 48 patients with acute ischaemic stroke (1:1) to receive nine 10-min sessions of either HRV biofeedback or sham biofeedback over 3 days in addition to standard stroke unit care. An investigator (P.O.) generated the allocation sequence using an online randomizer (randomizer.org) and used sequentially numbered containers to conceal the sequence until interventions were assigned. Recruitment of study participants was undertaken from November 2018 to August 2019. At baseline all patients underwent medical history assessment and physical examination including evaluation for neurological deficits as well as autonomic testing of neurocardiac, sudomotor and vasomotor function. The study intervention, HRV biofeedback, was performed over 10 min, three times a day for three consecutive days, starting at baseline in a single masked setting where patients were blinded to the group allocation (sham control vs. biofeedback). Autonomic testing was repeated immediately after the last biofeedback training. Assessment of severity of autonomic symptoms and functional impairment was performed at baseline and was repeated by means of a phone-based follow-up after 3 months. All autonomic and clinical assessments were performed by an investigator who was not blinded to treatment allocation (P.O.). Statistical analysis was performed by an investigator (K.B.) blinded to group allocation.

### Standard Stroke Unit Care

Patients with acute ischemic stroke received organized inpatient care provided by a multi-disciplinary staff of a comprehensive stroke unit as part of our tertiary stroke center.

### Study Intervention and Sham Control

Heart rate variability biofeedback was performed as previously described (9). Briefly, patients were instructed to breath rhythmically with a given breathing frequency to facilitate respiratory sinus arrhythmia. The defaulted metronomic breathing frequency of six breathing cycles/min led to a harmonized alternation of inspirational increase and expirational decrease of heart rate, and in consequence, to an increased amplitude of heart rate oscillations which can be displayed as increase in HRV. A software-based biofeedback system (Stress Pilot Manager^®^, BITsoft Health Systems GmbH, Bitburg, Germany) with associated ear pulse sensor was used for HRV biofeedback training with continuous assessment and visualization of HRV on the computer screen. In this visualization, a butterfly was hovering in the air and was ascending when HRV increased or descending when HRV decreased, respectively. Breathing instructions were given on the screen as a moving bar with upward movement signaling the patient to inhale and downward movement indicating to exhale. After having completed a test-training, patients underwent nine 10-min biofeedback training sessions over a period of 3 days. Frequency and duration of the intervention was determined in consideration of compatibility with standardized care procedures as part of stroke unit care as well as previously published intervention protocols and explorative studies of short-term HRV biofeedback (8, 9). An introductory session prior to each first biofeedback session was conducted to enhance adherence and each training session was visually monitored by the instructor (P.O.) for correct execution. Metronomic breathing was monitored using a respiratory belt transducer (TN1132/ST Respiratory Belt Transducer, ADInstruments, Castle Hill, Australia). Any violations of the intervention protocol were noted. After having completed the last biofeedback training sessions, participants were instructed to continue this breathing technique three times a day, however without any HRV measuring or biofeedback.

In the control group, patients underwent sham biofeedback sessions in an identical setting, duration and frequency as the active group except for the absence of any breathing instructions and real-time assessment or visualization of HRV. During sessions they were sitting in front of the computer screen and were looking at the image used to display HRV. Absent any input from the pulse sensor, no changes in HRV were displayed on the screen and the butterfly visualization remained at the same height.

### Assessment of Autonomic Functions

All biosignals derived from autonomic testing of neurocardiac, sudomotor and vasomotor function signals were processed by an artifact-filter and signal amplifier (Bridge Amp^®^ FE221, ADInstruments, Castle Hill, Australia) and were converted using a four-channel-digitizing polygraph (Power-Lab^®^, ADInstruments, Castle Hill, Australia). Analyses were carried out using the software package LabChart^®^ for Windows (ADInstruments, Castle Hill, Australia). In order not to interfere with stroke unit care all measurements took place in the patient's room of our stroke unit with a controlled room temperature of 21–25°C. Autonomic testing was performed in a semi-recumbent position after a 10-min lasting rest.

#### Assessment of Neurocardiac Function

We assessed HRV to evaluate neurocardiac autonomic function. A 3-channel electrocardiogram (MLA2503^®^ ADInstruments, Castle Hill, Australia) recorded cardiac electrical activity over two phases of 3 min each. In the first phase, patients were instructed to breathe casually, whereas in the second phase patients breathed at a fixed frequency of six per minute with an inspiration/expiration ratio of 1.5/1 as indicated by an audio signal to increase parasympathetic activity and thereby HRV (10). For both phases, casual and paced breathing, time domain analysis of HRV was undertaken by calculating the primary outcome measure SDNN, the standard deviation of all intervals between adjacent QRS complexes resulting from sinus node depolarizations (normal-to-normal intervals). In addition the predominantly parasympathetic time domain measure root mean square of successive differences between normal heartbeats (RMSSD) was calculated in both phases. Power spectral density analysis of HRV was performed using Fast-Fourier-Transformation as previously described to calculate spectral components, that is, total power, high frequency, low frequency and very low frequency (11). The low frequency/high frequency ratio was then calculated to estimate symapathovagal balance.

#### Assessment of Sudomotor Autonomic Function

The sympathetic skin response was measured to assess sudomotor function as previously desribed (12). Briefly, changes in skin conductance after sympathetic activation through sudden deep inspiration were quantified using two finger electrodes (MLT116F^®^, ADInstruments, Castle Hill, Australia). The maximum increase in amplitude following was calculated as a measure of the sympathetic sudomotor response.

#### Assessment of Vasomotor Autonomic Function

Cutaneous blood flow following sympathetic stimulation was measured using a plethysmograph (MLT1020PPG, ADInstruments, Castle Hill, Australia) equipped with an infrared photoelectric sensor as previously described (13). Briefly, a diode emitted infrared light (950 nm) which was partially absorbed while transmitting through the finger. The sensor received the reflected non-absorbed light, generating an electrical current, which was proportional to the amount of this reflected light and thereby indicated changes in tissue blood volume. Photoplethysmography was performed to qantify the vasoconstrictory response of cutaneous blood vessels at a depth of 2 mm to assess sympathetic vasomotor function following forced deep inspiration. The vasoconstrictory response was defined as blood flow at baseline minus the lowest value post deep inspiration over blood flow at baseline. Additionally, durations to 50% constriction and 50% redilatation of cutaneous vessels were calculated.

#### Assessment of Symptoms and Functional Impairment

Severity of symptoms attributed to the autonomic nervous system were assessed using the German translation of the Survey of Autonomic Symptoms (14, 15). The Total Symptom Impact Score (TIS) was calculated by summating the rated severity of individual item scores. Functional impairment was assessed using modified Rankin scale (mRS) (16). Functional impairment (mRS after 3 months) was expected to decrease in both groups of patients with acute ischaemic stroke and was assessed to provide pilot data for sample size calculation in follow up research to investigate the effects of HRV biofeedback on functional recovery. Severity of neurological deficits was assessed using National Institutes of Health Stroke Scale (NIHSS) as part of baseline characterization (17).

### Statistical Analysis

All statistical analyses were performed with STATA software (Version 12.1, StataCorp., College Station, TX). Following testing for normality and equality of variances, Student's *t*-test for independent samples, Wilcoxon rank-sum test, Chi-square test and Fisher's exact test were applied to compare baseline characteristics between groups, where applicable. The primary outcome was change of SDNN from baseline to post intervention under paced breathing. Further outcomes of clinical interest comprised RMSSD, spectral analysis parameters of HRV (total power, high frequency, low frequency, very low frequency, low frequency/high frequency ratio), parameters of autonomic sudomotor (sympathetic skin response) and vasomotor (vasoconstrictory response, duration to 50% constriction, duration to 50% redilatation) function, and autonomic symptom severity (TIS). Since autonomic data followed a non-normal distribution, the Wilcoxon signed-rank test was used for dependent within-subject comparisons and the Wilcoxon rank-sum test for independent between-subject comparisons. Data are expressed as median [interquartile range], mean (± standard deviation) or percentage according to type and distribution.

Due to the exploratory nature of this study no formal sample size calculation was undertaken. Sample size of this pilot study was determined based on a previous study of HRV biofeedback in patients with alcohol dependence showing cardiac autonomic improvement post intervention (18). Student's *t*-test for independent samples, Wilcoxon rank-sum test, Chi-square test, Fisher's exact test, Wilcoxon signed-rank test or McNemar's chi-square test were applied where applicable in the intention-to-treat population to compare outcomes with statistical significance set as *p*-value <0.05. While the datasets of our primary outcome and all autonomic tests were complete, missing data points in the 3-month follow up were considered missing at random and were treated using an available case analysis. All statistical analyses were performed by original assigned groups.

## Results

### Demographics and Baseline Characteristics

We included 48 patients [19 females, ages 65 (4.4), median (interquartile range)] with acute ischaemic stroke. The study was ended after complete recruitment of the predetermined sample size. All analyses were by original assigned groups. Patients in the HRV biofeedback group (*n* = 24) did not differ from those in the sham-biofeedback group (*n* = 24) with respect to sex, age, body mass index, alcohol consumption, cardiovascular comorbidities, medication, clinical baseline value, stroke etiology and acute treatment (Table 1).

**Table 1:** Demographics and baseline characteristics.

	**HRV biofeedback**	**Sham-Biofeedback**	*P-*value^*^
	**(*n* = 24)**	**(*n* = 24)**	
**Demographics**			
Females, *n* (%)	9 (37.5)	10 (41.7)	0.77
Age (years)	66 [19.5]	69.5 [17.5]	0.36
Weight (kg)	81.8 (± 14.4)	78.0 (± 14.1)	0.37
Height (cm)	173.3 (± 9.6)	171.4 (± 9.6)	0.49
Body mass index (kg/m^2^)	26.5 [6.4]	27.4 [4.3]	0.96
Juvenile stroke, *n* (%)	4 (16.7)	2 (8.3)	0.67
**Cardiovascular risk profile**			
Diabetes mellitus type I, *n* (%)	1 (4.2)	0 (0.0)	1.00
Diabetes mellitus type II, *n* (%)	3 (12.5)	8 (33.3)	0.17
Arterial hypertension, *n* (%)	16 (66.7)	17 (70.8)	0.76
Hyperlipidemia, *n* (%)	6 (25.0)	7 (29.2)	0.75
Smoking, *n* (%)	6 (25.0)	6 (25.0)	1.00
Alcohol consumption, *n* (%)	7 (29.2)	3 (12.5)	0.29
**Medication**			
Antidiabetic, *n* (%)	4 (16.7)	7 (29.2)	0.50
Antihypertensive: all, *n* (%)	15 (62.5)	14 (58.3)	0.77
Antihypertensive: beta-blocker, *n* (%)	5 (20.8)	7 (29.2)	0.74
Lipid lowering, *n* (%)	6 (25.0)	5 (20.8)	1.00
**Clinical baseline values**			
Heart rate (1/min)	79.2 (±15.0)	73.3 (±10.2)	0.12
Systolic blood pressure (mmHg)	132.3 (±15.8)	139.0 (±17.0)	0.16
Diastolic blood pressure (mmHg)	71.1 (±10.7)	72.7 (±10.4)	0.62
Baseline NIHSS	1.5 [2.0]	2.0 [4.0]	0.97
Baseline mRS	2.0 [1.0]	2.0 [2.0]	0.67
Baseline TIS	7.5 [7.0]	5.5 [7.5]	0.42
**Stroke etiology**			
TOAST classification			0.21
Large-artery atherosclerosis, *n* (%)	9 (37.5)	3 (12.5)	-
Cardioembolism, *n* (%)	4 (16.7)	2 (8.3)	-
Small-vessel occlusion, *n* (%)	4 (16.7)	6 (25.0)	-
Other determined etiology, *n* (%)	1 (4.2)	2 (8.3)	-
Undetermined etiology, *n* (%)	6 (25.0)	11 (45.8)	-
**Localization of ischemia**			
Anterior circulation [L], *n* (%)	7 (29.2)	7 (29.2)	1.00
Anterior circulation [R], *n* (%)	7 (29.2)	7 (29.2)	1.00
Posterior circulation, *n* (%)	10 (41.7)	10 (41.7)	1.00
**Acute treatment**			
Intravenous thrombolysis, *n* (%)	4(16.7)	3(12.5)	1.00
Endovascular treatment, *n* (%)	3(12.5)	1(4.2)	0.61

*Data are expressed as median [interquartile range], mean (± standard deviation) or percentage. NIHSS, National Institutes of Health Stroke Scale; mRS, National Institutes of Health Stroke Scale; TIS, survey of autonomic symptoms total symptom impact score; TOAST, trial of ORG 10172 in acute stroke treatment; R, right; L, left*.

* *p-value refers to between-group comparisons*.

### Missing Data, Safety and Adherence

**Figure 1:**
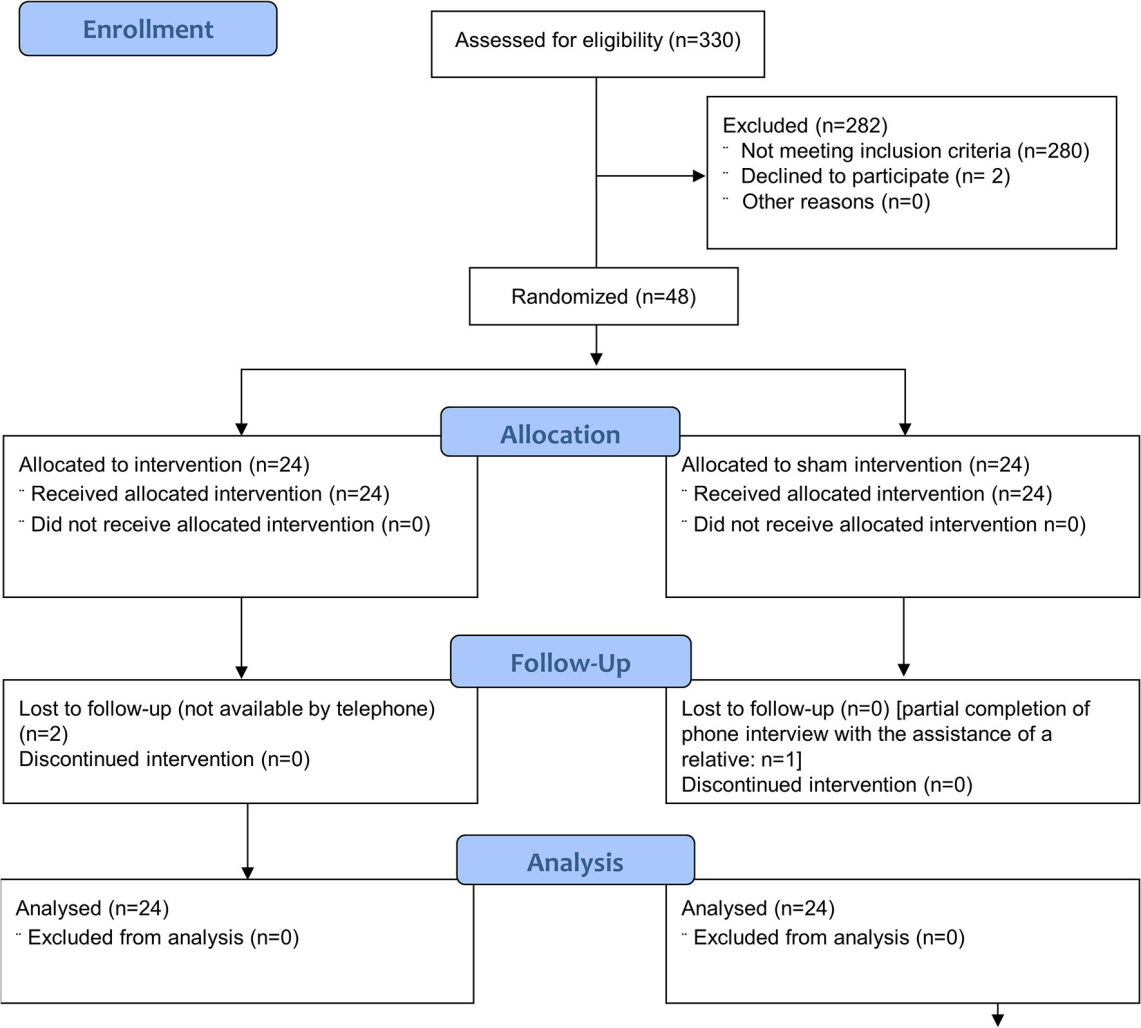
The Consolidated Standards of Reporting Trials (CONSORT) flow diagram of the progress through the phases of the parallel randomised sham controlled trial conducted [modified from (19)].

There were no missing data in the primary outcome and autonomic functions data set. Missing data were limited to two patients who were lost to the phone-based follow up assessment of TIS and mRS after 3 months and one patient with an incomplete 3-month follow up (study flow diagram: Figure 1).

The HRV biofeedback application and the sham intervention were well-tolerated by all study participants such that adherence was uncompromised throughout the study. In both study groups, initiation of intervention was achieved on the day of randomization in all patients. One patient reported temporary light dizziness during HRV biofeedback. Adherence was not compromised by the event and no other adverse events were noted. Randomization and initiation of the intervention was performed on the same day in all patients. The CONSORT checklist of information to include when reporting a randomized trial assessing non-pharmacologic treatments is shown in the Supplementary Material.

### Neurocardiac Function

**Figure 2:**
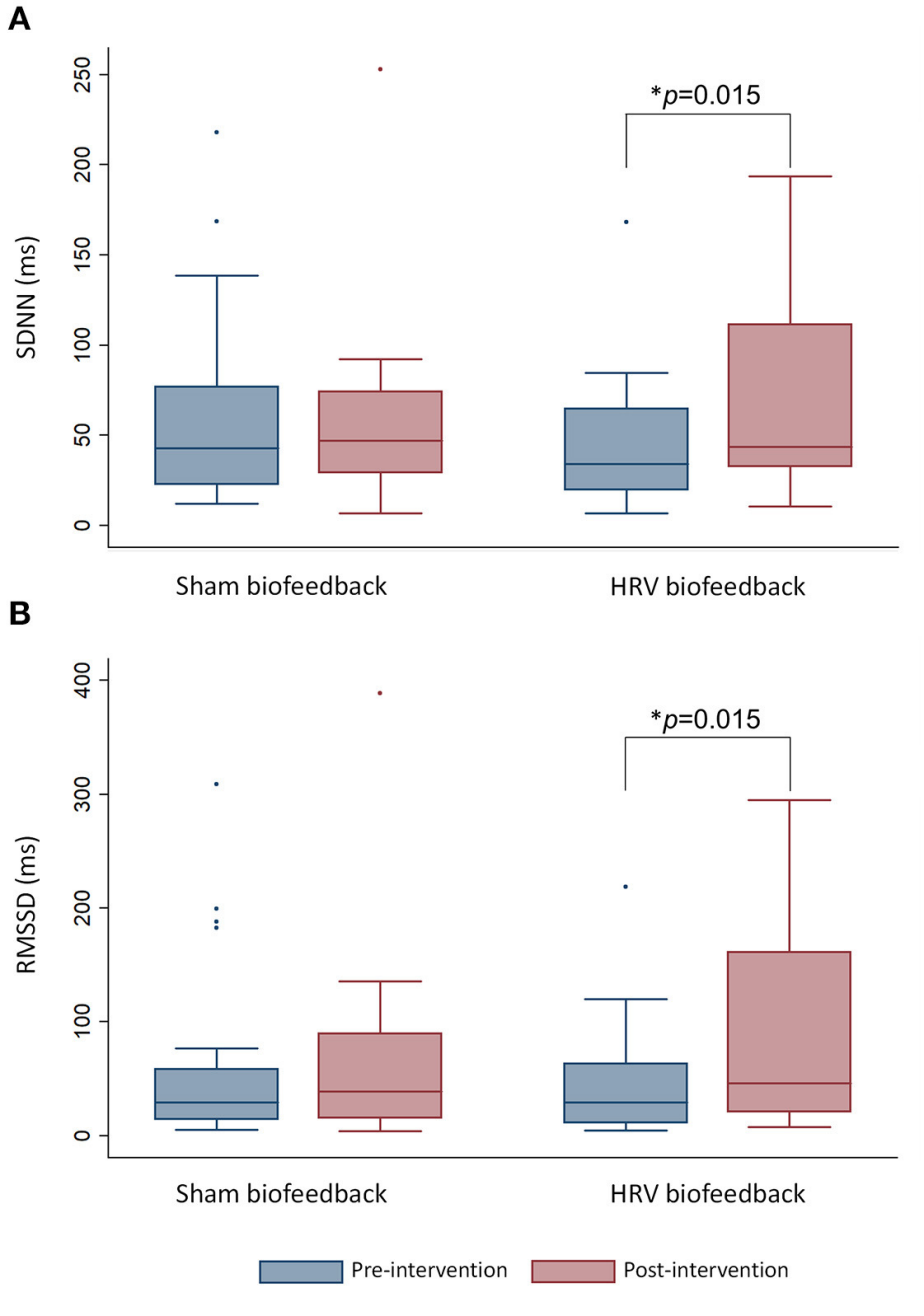
The box-and-whisker plot shows an increase in heart rate variability measured *via* **(A)** SDNN and **(B)** RMSSD under paced breathing after HRV biofeedback (*n* = 24) but not after sham biofeedback (*n* = 24). Whiskers indicate variability outside the upper and lower quartiles. The median is depicted as horizontal line. Outliers are plotted as individual points. *p-value refers to within-group comparisons.

Patients who received HRV biofeedback in addition to stroke unit care (*n* = 24) displayed an increase in HRV under paced breathing condition on time-domain analysis with elevated SDNN (Figure 2A) post-intervention compared to baseline, which was not noted after sham-biofeedback (*n* = 24). The predominantly parasympathetic HRV measure RMSSD was also elevated following HRV biofeedback but not sham biofeedback (Figure 2B).

There was neither such change when cardiac autonomic function was assessed during normal breathing *via* SDNN [HRV biofeedback, *n* = 24: 27.1 (43.5) baseline vs. 40.2 (30.8) ms post-intervention, *p* = 0.63; sham biofeedback, *n* = 24: 25.4 (28.2) ms baseline vs. 29.5 (33.5) post-intervention *p* = 0.46] nor *via* RMSSD [HRV biofeedback, *n* = 24: 18.6 (57.8) ms baseline vs. 34.0 (52.2) ms post-intervention, *p* = 0.65; sham biofeedback, *n* = 24: 17.9 (34.9) ms baseline vs. 24.5 (45.0) post-intervention *p* = 0.55].

Furthermore, analysis of spectral components of HRV under paced breathing conditions showed an increase in low frequency band and total power in patients who underwent HRV biofeedback but not in those who received the sham intervention.

In contrast, there were no such changes when spectral analysis of HRV was undertaken under normal breathing (Table 2).

**Table 2:** Measures of autonomic cardiac, sudomotor, and vasomotor function.

	**HRV biofeedback (*****n*** **=** **24)**			**Sham biofeedback (*****n*** **=** **24)**		
**Parameters**	**Baseline**	**Post-intervention**	*P*-value^*^	**Baseline**	**Post-intervention**	*P*-value^*^
**Cardiac autonomic function**						
*(HRV spectral components)*						
**Very low frequency (ms**^**2**^**)**						
Normal breathing Paced breathing	67.6 [273.1] 43.3 [213.5]	130.9. [227.4] 137.9 [276.9]	0.25 0.30	55.0 [249.0] 99.0 [409.3]	42.2 [305.1] 105.2 [301.4]	0.80 0.69
**Low frequency (ms**^**2**^**)**						
Normal breathing Paced breathing	129.7 [607.8] 484.8 [1941.4]	261.4 [591.8] 1471.3 [3329.9]	0.29 0.02	104.4 [436.7] 719.3 [1979.8]	56.2 [359.5] 499.0 [1739.2]	0.84 0.21
**High frequency (ms**^**2**^**)**						
Normal breathing Paced breathing	122.7 [824.1] 228.3 [870.8]	288.0 [840.4] 278.9 [5440.4]	0.63 0.09	124.7 [670.1] 143.6 [775.4]	142.2 [482.1] 172.3 [1164.3]	0.44 0.46
**Low frequency/high frequency ratio**						
Normal breathing Paced breathing	0.8 [1.8] 3.1 [8.9]	0.9 [2.1] 1.6 [9.8]	0.86 0.44	1.2 [2.0] 7.0 [8.9]	0.8 [1.1] 4.4 [7.9]	0.16 0.29
**Total Power (ms**^**2**^**)**						
Normal breathing Paced breathing	537.1 [1676.0] 1273.9 [3299.2]	1076.1[1647.5] 1771.5 [13038.8]	0.41 0.02	573.7 [1434.4] 1816.7 [4415.0]	823.2 [1320.6] 1780.2 [5589.4]	0.59 0.69
**Sudomotor function**						
Sympathetic skin response (μS)	2.5 [2.9]	1.4 [2.4]	0.19	2.1 [3.0]	1.0 [2.6]	0.06
**Vasomotor function**						
Duration to 50% constriction (s)	4.0 [9.3]	5.1 [9.3]	0.69	7.4 [7.2]	8.0 [9.7]	0.82
Duration to 50% redilatation (s)	2.8 [1.0]	2.2 [1.9]	0.13	2.7 [2.2]	2.4 [2.4]	0.63
Vasoconstrictory response (%)	22.5 [19.8]	20.5 [19.9]	0.29	18.3 [21.8]	23.6 [38.3]	0.14

*Data are expressed as median [interquartile range], HRV, heart rate variability; s, seconds; ms, millseconds; μS, microsievert*.

* *p-value refers to within-group comparisons*.

### Sudomotor and Vasomotor Autonomic Function

Assessment of sympathetic skin response neither revealed any changes of sudomotor autonomic function in patients with acute ischaemic stroke following HRV biofeedback training nor in those who have undergone sham biofeedback (Table 2). Adding HRV biofeedback or sham biofeedback to standard stroke unit care did not alter vasomotor function (Table 2).

### Autonomic Symptoms and Functional Impairment

The addition of HRV biofeedback to standard stroke unit care led to an alleviation of severity of autonomic symptoms 3 months post intervention, which was not seen after sham biofeedback [HRV biofeedback 3.5 (8.0) vs. 7.5 (7.0) baseline, *p* = 0.029; sham biofeedback: 4.0 (7.0) vs. 5.5 (7.5), *p* = 0.06]. As expected functional deficits measured after the intervention were decreased in both study groups [HRV biofeedback 0.0 (2.0) vs. 2.0 (1.0) baseline, *p* = 0.023; sham biofeedback: 1.0 (2.0) vs. 2.0 (2.0) baseline, *p* = 0.0005].

## Discussion

The major finding of this randomized sham-controlled pilot study is that HRV biofeedback might improve neurocardiac function and alleviate autonomic symptoms in patients with acute ischaemic stroke undergoing stroke unit care. The treatment was well-tolerated by stroke patients and its integration into the multidisciplinary setting of a comprehensive stroke unit was feasible. Improvement in parasympathetic function following HRV biofeedback was neither paralleled by improvement in measures of sympathetic function nor in sympathovagal balance, indicating a predominantly parasympathetic mechanism of action. Lastly, we were able to gather pilot data to provide a basis for calculating the sample size of follow-up research to confirm these findings and study the interventions' effects on functional recovery from acute ischaemic stroke.

Multiple clinical studies have shown that HRV biofeedback can alleviate neurocardiac dysfunction and improve clinical outcomes in neuropsychiatric and cardiovascular disorders, possibly mediated by augmented respiratory sinus arrhythmia triggering increased baroreflex gain and parasympathetic outflow (20, 21). This would explain why in previous controlled studies HRV biofeedback was able to counteract a shift of the autonomic balance toward the sympathetic nervous system and was therefore particularly efficacious in improving cardiac autonomic function in conditions that are associated with chronic sympathetic hyperactivity (parasympathetic withdrawal, respectively) such as depression, addiction to alcohol and posttraumatic stress disorder (18, 22–24). Notably, in these studies the beneficial effects of HRV biofeedback exceeded mere neurocardiac improvement but also translated into alleviation of clinical outcomes such as depressive symptoms and craving for alcohol. In line with these observations, we observed improvement in neurocardiac autonomic function and sustained alleviation of autonomic symptoms (8, 25). While an increase in the predominantly parasympathetic measures of neurocardiac function in response to HRV biofeedback has been observed consistently throughout previous studies in patients with psychiatric and cardiovascular disorders, the treatment's effects on the sympathetic nervous system remain poorly understood (21). A randomized controlled study in patients with alcohol dependence showed improvement of vasomotor but not sudomotor sympathetic function following HRV biofeedback. This observation contrasts our finding of unchanged vasomotor and sudomotor function following HRV biofeedback after acute ischaemic stroke, which might be explained by high inter-subject variability of sympathetic skin response (12, 18).

Spectral analysis of HRV showed an increase of total power after HRV biofeedback, consistent with an overall increase in HRV. Analysis also revealed an increase in low frequency, a HRV component considered to be determined by both branches of the autonomic nervous system. This could indicate a sympathetic component to the mechanism whereby the study intervention improved neurocardiac function but is in contrast with the absence of any changes in neurocardiac sympathovagal balance and non-cardiac sympathetic measures in our study. Alternatively, viewed in conjunction with the observed increase in SDNN and RMSSD after HRV biofeedback, these findings might support the previously reported superior precision of time domain over spectral analysis of frequency domains in the evaluation of neurocardiac function (26). This is also consistent with an ongoing controversy on the diagnostic value of spectral analysis, which has been fueled by accumulating evidence of the technique's limited precision in discriminating between sympathetic and parasympathetic HRV components (27). However, in our study, non-cardiac sympathetic function measures remained unaltered after HRV biofeedback consistent with a predominantly parasympathetic mechanism of action. This is also in line with our observation that changes in cardiac autonomic function became significant when HRV was assessed under paced breathing, a maneuver that stimulates parasympathetic outflow.

Feasibility of implementing HRV biofeedback into a standardized multidisciplinary stroke unit care as well as high tolerability of the treatment procedure in our study indicate that the treatment can be added to standard care of patients with acute ischaemic stroke to reduce neurocardiac dysfunction post-stroke without jeopardizing integrative care. A previous randomized study of HRV biofeedback enrolled patients who have experienced an ischemic stroke within 1 week from stroke onset also showed an increase in HRV after treatment compared to a control group that received no study intervention (28). However, patients were recruited at a neurological ward and it was not specified whether this ward employs multidisciplinary stroke unit care. Moreover, it was not reported if adverse events or tolerability were measured. However, the dropout rate was low (5 out of 40 included patients) in this study and most of the dropouts (3 out of 5) occurred because of refusing baseline assessment.

Although absolute differences in time domain measures of HRV were relatively small between patients treated with HRV biofeedback and sham control patients, they might be clinically meaningful since decreased HRV has shown to predict all cause-death and cardiovascular events with likely implication to cerebrovascular disease (29–32). However, due to the pilot nature of our study this finding needs confirmation in a larger population before a clear recommendation can derive from it.

Strengths of our study include its randomized control design and the assessment of non-cardiac vasomotor and sudomotor sympathetic function measures allowing detailed phenotyping of autonomic functional integrity. We were able to test the implementation of HRV biofeedback treatment within a state-of the art comprehensive stroke unit, underscoring the probable external validity of our observations. Our study is limited by the lack of a long-term follow up assessment of neurocardiac function. Therefore, we do not know if the observed increase of HRV after treatment would be sustained in later phases of rehabilitative therapy. However our results suggest alleviation of autonomic symptoms after HRV biofeedback, which was found to be sustained 3 months post-intervention, indicating a possible beneficial long-term effect on functional integrity the autonomic nervous system. We did not perform any analysis on possible effects of onset-to-randomization time or ischaemic damage to specific autonomic control centers on treatment effects because of the limited sized of our study population. However these associations will be investigated in a larger confirmatory study. Moreover, our study provides pilot data for a follow-up trial in a larger study population, which will also include assessment of the treatment's efficacy in facilitating functional recovery from stroke. In contrast to the majority of previous studies on HRV biofeedback, we included a sham control to minimize placebo effect, a strategy that was shown to increase effect sizes compared to inactive control conditions (33).

## Conclusions

In conclusion, HRV biofeedback might be a feasible and efficacious tool to counteract neurocardiac dysfunction and alleviate autonomic symptoms following acute ischaemic stroke. However, broad clinical implementation of the treatment requires follow up research in a large study population of patients with acute ischaemic stroke to understand whether modulation of autonomic neurocardiac function may translate into improved functional recovery and prevention of recurrent cerebrovascular events.

## Data Availability Statement

The raw data supporting the conclusions of this article will be made available by the authors, without undue reservation.

## Ethics Statement

The study was approved by the ethical review committee of Technical University of Dresden (Die Ethikkommission an der TU Dresden (study reference number: EK389102018, Office for Human Research Protections IRB reference: IRB00001473). Written and oral informed consent was obtained from each study participant. Clinicaltrials.gov registration: NCT03865225.

## Author Contributions

TS: supervision of data acquisition, drafting of the manuscript, study concept and design, analysis and interpretation of data. PO: acquisition of data (major role), revising manuscript for content, and interpretation of data. AS and JB: revising manuscript for content and interpretation of data. ES, MA, and L-PP: acquisition of data (supporting role) and revising manuscript for content. GR: analysis and interpretation of data and revising manuscript for content. HR and VP: study concept and revising manuscript for content. KB: study concept, analysis of data, and revising manuscript for content. All authors contributed to the article and approved the submitted version.

## Conflict of Interest

The authors declare that the research was conducted in the absence of any commercial or financial relationships that could be construed as a potential conflict of interest.

## Supplementary Material

The Supplementary Material for this article can be found online at: https://www.frontiersin.org/articles/10.3389/fneur.2021.669843/full#supplementary-material. Page numbers in the CONSORT checklist refer to the manuscript draft.
